# Factors associated with inadequate blood pressure control among hypertensive individuals living in a Quilombola Community: a cross-setional study, Brazil, 2017-2018

**DOI:** 10.1590/S2237-96222025v34e20240556.en

**Published:** 2025-10-03

**Authors:** Randson Souza Rosa, Junior Santos Menezes, Edson Zangiacomi Martinez, Rita Narriman Silva de Oliveira Boery, Ícaro José Santos Ribeiro, Jean Carlos Zambrano Contreras, José de Bessa, Ricardo Franklin de Freitas Mussi, Sávio Luiz Ferreira Moreira, Gisele Silveira Lemos, Isleide Santana Cardoso Santos

**Affiliations:** 1 Universidade Estadual do Sudoeste da Bahia, Departamento de Saúde, Jequié, BA, Brazil; 2 Universidade Estadual de Feira de Santana, Departamento de Saúde, Feira de Santana, BA, Brazil; 3 Universidade de São Paulo, Faculdade de Medicina de Ribeirão Preto, Ribeirão Preto, SP, Brazil; 4 Universidade Estadual de Santa Cruz, Departamento de Saúde, Ilhéus, BA, Brazil; 5 Universidade do Estado da Bahia, Departamento de Educação, Caetité, BA, Brazil

**Keywords:** Arterial Pressure, Risk Factors, Quilombola Communities, Black People, Cross-Sectional Studies, Presión Arterial, Factores de Riesgo, Quilombola, Población Negra, Estudios Transversales

## Abstract

**Objective:**

To identify the factors associated with the prevalence of inadequate blood pressure control among hypertensive residents of a Quilombola community in Northeastern Brazil.

**Methods:**

A cross-sectional epidemiological study with the participation of residents of Quilombo do Barro Preto (Jequié, Bahia) aged 35 to 79 years. Blood pressure control was classified as inadequate for systolic blood pressure ≥140 mmHg and/or diastolic blood pressure ≥90 mmHg. Medication adherence was assessed according to the Morisky Medication Adherence Scale. Using log-binomial regression, prevalence ratios (PR) adjusted for sex and age were estimated with 95% confidence intervals (95%CI) to compare differences between the prevalence of inadequate blood pressure control among interest groups. Comparisons between means were based on Student’s t-Tests and analysis of variance.

**Results:**

Among the 300 participants, 71.7% were women, 49.3% self-identified as Black, 41.0% Brown, and 39.7% had incomplete elementary education. Systolic blood pressure was associated with age, with a higher average among people over 65 years of age, while the average diastolic blood pressure was higher among people up to 55 years of age. The prevalence of inadequate pressure control was 66.3% (95%CI 60.7; 71.7), being higher in people with type 2 diabetes (PR1.28; 95%CI 1.09; 1.51) and low adherence to medications (PR1.27; 95%CI 1.01; 1.59).

**Conclusions:**

The Quilombola population presents difficulties in controlling hypertension, with a high prevalence of uncontrolled blood pressure, especially among people with type 2 diabetes and low adherence to drug treatment.

## Resumen

**Objetivo:**

Identificar los factores asociados a la prevalencia del control inadecuado de la presión arterial en personas hipertensas residentes en una comunidad quilombola en el Nordeste de Brasil.

**Métodos:**

Estudio epidemiológico de diseño transversal con la participación de residentes del Quilombo de Barro Preto (Jequié, Bahía), con edades entre 35 y 79 años. El control de la presión arterial se clasificó como inadecuado cuando la presión arterial sistólica era ≥140 mmHg y/o la presión arterial diastólica ≥90 mmHg. La adherencia al tratamiento medicamentoso fue evaluada mediante la *Morisky Medication Adherence Scale*. Se estimaron razones de prevalencia (RP) ajustadas por sexo y edad con intervalos de confianza del 95% (IC95%) utilizando regresión log-binomial, para comparar diferencias en las prevalencias de control inadecuado de la presión entre los grupos de interés. Las comparaciones de medias se realizaron con pruebas t de Student y análisis de varianza.

**Resultados:**

Entre los 300 participantes, el 71,7% eran mujeres, el 49,3% se autodeclararon negros, el 41,0% pardos y el 39,7% tenía nivel de escolaridad primaria incompleta. La presión arterial sistólica se asoció con la edad, con una media mayor entre personas mayores de 65 años; por otro lado, la media de la presión arterial diastólica fue mayor entre personas de hasta 55 años. La prevalencia de control inadecuado de la presión fue del 66,3% (IC95%: 60,7; 71,7), siendo mayor entre personas con diabetes tipo 2 (RP 1,28; IC95%: 1,09; 1,51) y con baja adherencia a los medicamentos (RP 1,27; IC95%: 1,01; 1,59).

**Conclusione:**

s: La población quilombola presenta dificultades en el control de la hipertensión arterial, con una alta prevalencia de descontrol de la presión, especialmente entre personas con diabetes tipo 2 y baja adherencia al tratamiento medicamentoso.

**Ethical aspects**

This research respected ethical principles, having obtained the following approval data:

Research Ethics Committee: Faculdade Independente do Nordeste

Opinion number: 2,015,327

Approval date: 13/4/2017

Certificate of Submission for Ethical Appraisal: 66705617.2.0000.5578

Informed Consent Form: Obtained from all participants prior to data collection.

## Introduction

High blood pressure is one of the most common chronic diseases in the world and a major challenge for public health. In 2019, it was the leading cause of death among women and the second among men, accounting for about 11 million deaths (1). Afro-descendants are especially affected, presenting higher prevalence and morbidity and mortality rates compared to other ethnic-racial groups (2).

Afro-descendant communities in Brazil experience an epidemiological transition marked by an urban lifestyle, which increases the risk of cardiometabolic diseases, especially among women, who have a more significant combination of risk factors compared to men (3). In Vitória da Conquista, Bahia, 45.4% of Quilombola residents had hypertension, associated with factors such as age, low education level, economic class, insecurity in the neighborhood, sedentary lifestyle, and obesity (4). Black people, especially low-income people, face greater barriers to treatment, such as a lack of social support, difficulty accessing healthy food and physical activities, and financial limitations. Uncontrolled hypertension increases the risk of serious complications and increases the burden on health systems in developing countries (5,6).

Evidence from the Reasons for Geographic and Racial Differences in Stroke (REGARDS) study highlighted that social determinants, such as low income, low education level, areas with a shortage of health professionals, and high poverty, increase the risk of uncontrolled blood pressure in black adults (7). The Bambuí-Epigen cohort study identified that socioeconomic status-especially education and family income-is the main non-biological factor associated with systolic blood pressure levels and hypertension control, regardless of age, gender, and other health indicators (8). This same study showed that blood pressure control in people of African descent is influenced by socioeconomic, cultural, behavioral, and biological factors, with socioeconomic position - such as low education level and income - being more determinant for hypertension control than the African genomic ancestry itself (8). On the other hand, the population-based cross-sectional study that used data from the Quilombola Communities of Vitória da Conquista (*Comunidades Quilombolas de Vitória da Conquista* - COMQUISTA) project showed that Quilombola communities have a high prevalence of prehypertension and hypertension associated with males, low education level, diabetes, and overweight/ obesity (9).

Quilombola communities, usually located in rural areas, are formed by descendants of Africans with a strong cultural and historical heritage of resistance who share knowledge, health practices, and ways of life inherited from their ancestors (10). However, they face multiple vulnerabilities, such as low income, lack of basic sanitation, precarious housing, difficulties in accessing health and public services, as well as food and nutritional insecurity (11).

The control of hypertension among black people is disproportionately affected by behavioral, environmental, social, and structural factors, which shows that increased awareness and treatment rates, although important, are not enough. Public health strategies must be adapted to the specific needs of these individuals and their communities (12). Low adherence to treatment is one of the main challenges for blood pressure control among black people, and non-adherence to antihypertensive medication is a significant barrier that directly contributes to racial disparities in cardiovascular morbidity and mortality (13). Epidemiological studies are important to map the diseases that most affect Quilombola populations, guiding public policies more objectively to meet their specific demands (14,15). 

Hypertension is one of the main causes of morbidity and mortality in Brazil, with an even greater impact on socially vulnerable populations. In the context of Quilombola communities - marked by historical inequalities, structural racism, and limited access to health services - inadequate pressure control represents an important public health challenge. Despite the high burden of cardiovascular diseases among Afro-descendants, there is a gap in studies focused specifically on the factors associated with hypertension control in these communities, especially in Northeastern Brazil.

The present study is justified by the need to show contextually relevant results on the determinants of blood pressure imbalance among the hypertensive Quilombola population, considering individual, socioeconomic, and health-related factors. The research is innovative for integrating different aspects (age group and adherence to medication use) in a population historically invisible in traditional epidemiological studies and policies.

By identifying the main factors associated with inadequate blood pressure control in this group, this study may support the formulation of more equitable and culturally sensitive strategies for coping with cardiovascular diseases in black populations, contributing both to the reduction of health inequities and the effectiveness of the National Comprehensive Health Policy for the Black Population (*Política Nacional de Saúde Integral da População Negra* - PNSIPN). Therefore, the objective of this article was to identify the factors associated with the prevalence of inadequate blood pressure control in hypertensive residents of a Quilombola community in Northeastern Brazil.

## Methods

### Study design 

This is a cross-sectional epidemiological study. 

### Setting

The study was conducted in the Quilombola Community of Barro Preto, located in the Caixa D’Água neighborhood, within the coverage area of the Odorico Motta Health Center, located in the urban area of Jequié, in the Southwest Region of Bahia.

The community is officially recognized as a Quilombola community and was certified by the Palmares Cultural Foundation (*Fundação Cultural Palmares*) on February 14, 2007, under registration No. 01420.000313/2007-64. This recognition was ratified by Ordinance No. 104/2016, published in the Federal Official Gazette of Brazil on May 20, 2016 (15).

The community covers 26 streets and two squares, has approximately 2,066 houses, where about 1,262 families reside, totaling a population of approximately 7,130 people. 

The selection of this community is justified by the fact that it is home to a population of Quilombola individuals who preserve traditional knowledge, in a region located within an urban area.

### Participants

The research population corresponded to the number of records of people diagnosed with systemic arterial hypertension (SAH) registered at the Family Health Center of the Quilombo do Barro Preto community, located in Jequié, Bahia (400 hypertensive patients), using antihypertensive medications, of both sexes, aged between 35 and 79 years. All these individuals are self-identified Quilombolas, and the interviews were conducted in their homes by a previously trained team. The survey was conducted between November 2017 and March 2018.

### Research instruments

The study used questions from the instrument “satisfaction of hypertensive users with the services of the primary health care network”, validated for use in hypertensive adults served in services in Brazil (16). This instrument has two parts. The first contains a space for data collection, such as blood pressure, anthropometric, and sociodemographic data. The second part addresses risk factors and comorbidities, whose information was obtained in a self-reported manner. To assess medication adherence, the Portuguese version of the Morisky Medication Adherence Scale (MMAS-8, translated as the *Escala de Adesão Terapêutica de Morisky*) was applied, which has been adapted and validated for the Portuguese language (17). 

Of the total number of hypertensive Quilombolas identified, 25 were initially excluded due to absence or change of residence. After the interviews, another 75 were excluded for not having performed the blood collection or for not having completed the questionnaires. Therefore, the final sample consisted of 300 participants, who met the eligibility criteria and completed all stages of data collection.

### Data source and measurement

The first stage of data collection included the application of questionnaires, followed by the evaluation of body composition and the measurement of blood pressure for clinical evaluation. Blood pressure was measured twice in a quiet environment, the first being performed 10 minutes after the start of the clinical evaluation and the second five minutes later, according to the recommendations of the Brazilian Guidelines for Arterial Hypertension (*Diretrizes Brasileiras de Hipertensão Arterial*) (18). For the measurement, an Omron digital sphygmomanometer (model HEM-7113) was used, with the cuff adjusted according to the size and shape of the patient’s arm, validated and recommended by the Brazilian Society of Hypertension (*Sociedade Brasileira de Hipertensão*) (19). During these measurements, participants emptied their bladder and positioned their left arm at heart level. All measurements were performed by professionals previously trained to work in home-based data collection. For data analysis, the mean between the first and second blood pressure measurements was considered.

Regarding the measurement of body composition, including weight (in kg), height (in cm), and waist circumference (in cm), standardized anthropometric evaluation techniques were used. An Omron digital bioimpedance scale (model HBF-514c) was used to assess body mass, with participants wearing light clothing and positioned in the center of the device. To measure height, a portable Sanny stadiometer Personal Caprice (model ES2060) was used, with a measuring field of 210 cm. Participants were barefoot, with their feet placed together. Waist circumference was measured using a non-elastic, flexible measuring tape (Sanny brand, model SN-4010, without a locking mechanism), with participants in the supine position.

The cut-off points for predicting obesity of waist circumference equal to or greater than 102 cm for men and equal to or greater than 88 cm for women were adopted, as established by the criteria of the National Cholesterol Education Program (NCEP) III (20). Based on weight and height measurements, overweight and obesity classifications were determined using the body mass index (BMI), calculated by dividing weight by height squared. According to the guidelines of the World Health Organization (WHO) of 1995, participants were classified as overweight (overweight and obesity) when the body mass index was equal to or greater than 25 kg/m2, and as eutrophic or normal when less than 25 kg/m2 (21).

The second stage was conducted at the local Family Health Center, where trained technicians provided by the partner laboratory of the Municipal Health Department collected blood for analysis of the lipid profile of the study participants.

### Variables 

For data analysis, the dependent variable was blood pressure control, classified as uncontrolled (inadequate) blood pressure when systolic blood pressure was greater than or equal to 140 mmHg and/or diastolic blood pressure was greater than or equal to 90 mmHg, and adequate blood pressure control, indicated by blood pressure readings lower than 140/90 mmHg (19). The independent variables were: gender (male or female), age group (up to 55 years old, 56-65 years old or over 65 years old), race/skin color (Black, Brown, White, Asian or Indigenous), education (illiterate, literate, incomplete elementary education, complete elementary education, incomplete high school, complete high school or incomplete higher education), family/marital status (lives with partner, lives with relatives or lives alone), type 1 diabetes (yes, no or does not know), type 2 diabetes (yes, no or does not know), smoking (yes or no), alcoholism (yes, no or no answered), physical inactivity (yes or no), overweight (no; yes; not answered), acute myocardial infarction (yes or no), other coronary heart disease (yes, no or unknown), cerebrovascular accident (yes or no), and chronic kidney disease (yes, no or unknown). 

The MMAS-8 is composed of eight items, and each one is offered the answer options: “never”, “sometimes”, “often”, and “always”. Therapeutic adherence is then assessed by the sum of the “no” responses (1 point each). The adherence ratings are: high (eight points), medium (6 to <8 points), and low (<6 points).

### Bias control

The independent variables were collected through self-report. Therefore, there may be recall bias as well as bias due to a lack of understanding or comprehension regarding the questions asked or the diagnosis of any comorbidity.

### Statistical methods

For data description, means and standard deviations were calculated for quantitative variables, and absolute (n) and relative (%) frequencies were obtained for categorical variables. The percentage of individuals with inadequate blood pressure control was estimated with 95% confidence intervals (95%CI), using the Clopper-Pearson method (22), implemented in the DescTools package of R software, version 4.3.2. Prevalence ratios (PR) adjusted for sex and age were used to measure the association between inadequate blood pressure control and the independent variables. These measures and their corresponding 95%CI were estimated using log-binomial regression models (23), considering sex and age as confounding variables. The logbin package in R software was used to fit these models (23).

The Student’s t-test for independent samples was used to compare the mean ages of individuals classified as having uncontrolled and controlled blood pressure. Analysis of variance (with tests based on Type III sums of squares) was used to compare the population means of systolic and diastolic blood pressure across different subgroups, with age and sex as covariates. 

These procedures considered a significance level of 0.05 and were appropriate for the presence or absence of homoscedasticity (assessed by Bartlett’s test). At the same time, assumptions of residual normality, symmetry, and the influence of outliers were checked using appropriate plots (boxplots and normal probability plots). The database was stored in Mendeley Data (24).

## Results

Inadequate blood pressure control was prevalent in (n=199; 66.3%) (95%CI 60.7; 71.7) of the participants. The age distribution of participants with inadequate and adequate blood pressure control was similar, with mean ages of 60.3 years and 59.5 years, respectively (standard deviations (SD) of 11.2 years and 10.7 years, respectively), with no evidence of differences between the population means (p-value=0.566, according to the Student’s t-test) (Table 1)

**Table 1:** Prevalence ratios and 95% confidence intervals (95%CI) adjusted for sex and age of inadequate blood pressure control according to the study variables. Brazil, 2018 (n=300)

		Inadequate blood pressure control		Prevalence Ratio^a^
Variables	n (%)	n	% (95%CI)	% (95%CI)
Total	300 (100)	199	66.3 (60.7; 71.7)	-
Sex				
Female	215 (71.7)	140	65.1 (58.3; 71.5)	Reference
Male	85 (28.3)	59	69.4 (58.5; 79.0)	1.03 (0.86; 1.22)
**Age group** (years)				
Up to 55	106 (35.4)	73	68.9 (59.1; 77.5)	Reference
56-65	94 (31.2)	54	57.4 (46.8; 67.6)	0.84 (0.67; 1.04)
Over 65 years	100 (33.4)	72	72.0 (62.1; 80.5)	1.04 (0.87; 1.24)
**Race/skin color**				
Black	148 (49.4)	101	68.2 (60.1; 75.6)	Reference
Brown	123 (41.0)	80	65.0 (55.9; 73.4)	0.95 (0.81; 1.13)
White	24 (8.0)	15	62.5 (40.6; 81.2)	0.87 (0.63; 1.21)
Asian	4 (1.3)	2	50.0 (6.8; 93.2)	^b^
Indigenous	1 (0.3)	1	-	^b^
**Education level**				
Illiterate	88 (29.3)	65	73.9 (63.4; 82.7)	Reference
Literate	38 (12.7)	27	71.1 (54.1; 84.6)	0.96 (0.76; 1.22)
Incomplete elementary education	119 (39.8)	75	63.0 (53.7; 71.7)	0.84 (0.69; 1.02)
Complete elementary education	25 (8.3)	15	60.0 (38.7; 78.9)	0.74 (0.51; 1.06)
Incomplete high school	12 (4.0)	8	66.7 (34.9; 90.1)	0.83 (0.54; 1.28)
Complete high school	16 (5.3)	9	56.2 (29.9; 80.2)	0.71 (0.45; 1.28)
Complete higher education	2 (0.6)	0	-	^b^
**Family/marital status**				
Lives with a partner	176 (58.7)	117	66.5 (59.0; 73.4)	Reference
Lives with relatives	96 (32.0)	59	61.5 (51.0; 71.2)	0.93 (0.77; 1.13)
Lives alone	28 (9.3)	23	82.1 (63.1; 93.9)	1.16 (0.93; 1.45)
Smoking				
No	272 (90.7)	179	65.8 (59.8; 71.4)	Reference
Yes	28 (9.3)	20	71.4 (51.3; 86.8)	1,11 (0,87; 1,41)
**Alcoholism**				
No	248 (82.7)	165	66.5 (60.3; 72.4)	Reference
Yes	48 (16.0)	31	64.6 (49.5; 77.8)	0.93 (0.73; 1.18)
Not answered	4 (1.3)	3	-	-
**Physical inactivity**				
No	150 (50.0)	101	67.3 (59.2; 74.8)	Reference
Yes	150 (50.0)	98	65.3 (57.1; 72.9)	0.96 (0.82; 1.13)
**Medication adherence**				
High	73 (24.3)	42	57.5 (45.4; 69.0)	Reference
Medium	117 (39.0)	76	65.0 (55.6; 73.5)	1.12 (0.89; 1.41) ;
Low	110 (36.7)	81	73.6 (64.4; 81.6)	1.27 (1.01; 1.59)
**Type 1 diabetes**				
No	211 (70.3)	145	68.7 (62.0; 74.9)	Reference
Yes	6 (2.0)	4	66.7 (22.3; 95.7)	0.91 (0.51; 1.61)
Unknown	83 (27.7)	50	60.2 (48.9; 70.8)	0.88 (0.73; 1.07)
**Type 2 diabetes**				
No	147 (49.0)	92	62.6 (54.2; 70.4)	Reference
Yes	71 (23.7)	58	81.7 (70.7; 89.9)	1.28 (1.09; 1.51) d
Unknown	82 (27.3)	49	59.8 (48.3; 70.4)	0.96 (0.78; 1.19)
Overweight				
No	101 (33.7)	69	68.5 (58.3; 77.2)	Reference
Yes	193 (64.3)	127	65.8 (58.6; 72.5)	0.99 (0.84; 1.78)
Not answered	6 (2.0)	3	-	
**Acute myocardial infarction**				
No	293 (97.7)	194	66.2 (60.5; 71.6)	Reference
Yes	7 (2.3)	5	71.4 (29.0; 96.3)	1.09 (0.66; 1.79)
**Other coronary heart diseases**				
No	272 (90.7)	185	68.0 (62.1; 73.5)	Reference
Yes	26 (8.7)	13	50.0 (29.9; 70.1)	0.74 (0.50; 1.09)
Unknown	2 (0.6)	1	-	
**Cerebrovascular accident**				
No	284 (94.7)	189	66.5 (60.7; 72.0)	Reference
Yes	16 (5.3)	10	62.5 (35.4; 84.8)	0.90 (0.59; 1.36)
**Chronic kidney disease**				
No	204 (68.0)	133	65.2 (58.2; 71.7)	Reference
Yes	20 (6.7)	14	70.0 (45.7; 88.1)	1.07 (0.79; 1.44)
Unknown	76 (25.3)	2	-	-

^a^Prevalence ratios adjusted for sex and age; ^b^Not estimated due to small sample size in the corresponding category.

As shown in Table 1, the sociodemographic profile of the sample was composed predominantly of individuals: female (n=215; 71.7%), self-declared black (n=148; 49.3%) and Brown (n=123; 41.0%), aged up to 55 years (n=106; 35.4%), with incomplete elementary education (n=119; 39.7%), and living with a partner (n=176; 58.7%). 

Regarding lifestyle (Table 1), smoking was reported by 28 participants (9.2%), 48 participants (16%) reported alcohol consumption, 150 participants (50.0%) declared physical inactivity, while 110 participants (36.7%) and 117 participants (39%) showed, respectively, low and medium medication adherence.

Regarding comorbidities (Table 1), 6 participants (2.0%) reported type 1 diabetes, 71 participants (23.7%) reported type 2 diabetes, overweight was prevalent in 193 participants (64.3%), myocardial infarction in 7 participants (2.3%), other coronary heart disease in 26 participants (8.7%), stroke in 16 participants (5.3%), and chronic kidney disease in 20 participants (6.7%).

Considering the entire sample, the mean systolic and diastolic blood pressures were 150.3 mmHg (standard deviation [SD] 24.8 mmHg) and 86.4 mmHg (SD 13.3 mmHg), respectively (Table 2).

**Table 2:** Mean values and standard deviations (SD) of systolic and diastolic blood pressure according to study variables. Bahia, Brazil, 2018 (n=300).

		Systolic blood pressure (mmHg)		Diastolic blood pressure (mmHg)	
Variables	n	Average (SD)	p-value^a^	Average (SD)	p-value^a^
Total	300	150.3 (24.8)	-	86.4 (13.3)	-
Sex			0.920		0.623
Female	215	150.2 (25.3)		86.4 (13.2)	
Male	85	150.3 (23.4)		86.6 (13.7)	
**Age group** (years)			0.019		<0.001
Up to 55	106	150.6 (26.1)		92.3 (14.3)	
56-65	94	145.0 (23.8)		83.1 (12.1)	
Over 65 years	100	154.9 (23.4)		83.3 (11.2)	
**Race/skin color**			0.886		0.59
Black	148	150.7 (26.1)		87.3 (14.8)	
Brown	123	149.5 (23.4)		86.1 (11.8)	
White	24	150.4 (25.1)		84.4 (12.3)	
Asian	4	153.3 (19.7)		82.0 (6.0)	
**Education level**			0.020		0.453
Illiterate	88	158.2 (25.8)		85.3 (13.4)	
Literate	38	152.6 (28.1)		84.4 (12.1)	
Incomplete elementary education	119	146.7 (22.8)		87.5 (13.2)	
Complete elementary education	25	146.8 (18.1)		87.2 (11.3)	
Incomplete high school	12	140.7 (23.9)		90.7 (16.2)	
Complete high school	16	143.6 (26.0)		87.2 (17.1)	
Complete higher education	2	128.3 (8.8)		70.8 (13.8)	
**Family/marital status**			0.529		0.698
Lives with a partner	176	149.0 (24.0)		87.0 (12.6)	
Lives with relatives	96	151.0 (24.3)		85.5 (14.6)	
Lives alone	28	156.4 (26.0)		86.3 (13.3)	
Smoking					
No	272	150.2 (25.1)	0.864	86.5 (13.3)	0.794
Yes	28	151.2 (21.8)		85.6 (13.8)	
**Alcoholism**			0.188		0.382
No	248	151.1 (24.7)		85.8 (13.2)	
Yes	48	145.2 (23.7)		89.4 (14.0)	
**Physically inactive**			0.646		0.442
No	150	150.7 (24.2)		87.4 (13.4)	
Yes	150	149.9 (25.4)		85.5 (13.3)	
**Medication adherence**			0.015		0.032
Low	110	154.3 (26.5)		89.8 (14.4)	
Medium	117	149.8 (24.5)		85.2 (12.4)	
High	73	145.1 (21.5)		83.4 (12.1)	
**Type 1 diabetes**			0.451		0.789
No	211	151.2 (24.8)		86.7 (12.8)	
Yes	6	158.1 (24.7)		84.2 (9.4)	
Unknown	83	147.5 (24.6)		85.8 (14.8)	
**Type 2 diabetes**			0.081		0.738
No	147	149.1 (24.6)		86.6 (13.4)	
Yes	71	156.0 (24.5)		86.9 (11.2)	
Unknown	82	147.3 (24.7)		85.8 (14.9)	
Overweight			0.162		0.267
No	101	153.3 (24.7)		87.1 (13.2)	
Yes	193	148.7 (24.4)		86.2 (13.5)	
**Acute myocardial infarction**			0.998		0.441
No	293	150.3 (24.7)		86.6 (13.3)	
Yes	7	151.1 (30.1)		81.4 (15.4)	
**Other coronary heart diseases**			0.064		0.074
No	272	151.2 (24.9)		86.8 (13.5)	
Yes	26	141.4 (22.2)		82.7 (10.6)	
**Cerebrovascular accident**			0.745		0.287
No	284	150.1 (24.3)		86.3 (13.1)	
Yes	16	152.8 (31.3)		88.9 (16.9)	
**Chronic kidney disease**			0.982		0.803
No	204	149.3 (24.7)		85.7 (13.6)	
Yes	20	149.3 (22.6)		86.5 (10.5)	

^a^p-value obtained from analysis of variance (Type III tests), adjusted for sex and age.

A significant difference in mean systolic and diastolic blood pressure was identified according to age group (Table 2). In this regard, the mean systolic blood pressure was higher in the group aged over 65 years, while the mean diastolic blood pressure was higher among those under 55 years. 

## Discussion

This study highlights the vulnerability of a Quilombola community by identifying that two-thirds of individuals with hypertension have inadequate blood pressure control. The frequency of individuals in this condition is similar between sexes, shows no association with age, and remains high even among those with higher education levels. This underscores the importance of the Brazilian National Health System (*Sistema Único de Saúde* - SUS) and public pharmaceutical assistance policies, given that a significant number of individuals in Quilombola communities live in economically disadvantaged conditions and, in many cases, do not have sufficient income to maintain medication use unless it is provided by the public system (12). 

However, even among individuals with high medication adherence, a relatively high frequency of inadequate blood pressure control is observed (57.5%). Non-pharmacological lifestyle interventions should be encouraged among non-hypertensive individuals as preventive measures against the development of the disease, and, for those with established hypertension, as adjuvant therapy to reduce the risk of developing other comorbidities and to minimize the need for medications (25). These interventions include regular physical activity, weight control, smoking cessation, stress reduction, and prevention of excessive alcohol consumption (26). 

Although factors such as smoking, alcohol consumption, overweight, and physical inactivity did not show a direct association with blood pressure control, the high prevalence of overweight and physical inactivity suggests the need for ongoing monitoring and health interventions. In addition, a substantial proportion of participants reported type 2 diabetes, which was associated with a higher frequency of inadequate blood pressure control. Managing multiple health aspects in individuals with diabetes can hinder treatment adherence, highlighting the importance of good clinical practices to reduce complications and improve quality of life. Hypertension and diabetes are considered co-responsible for the main causes of mortality and hospitalizations in Brazil (27), and the coexistence of type 2 diabetes with hypertension can further complicate blood pressure management, requiring a more rigorous and complex therapeutic regimen. 

Blood pressure increases with age in a non-linear manner: diastolic pressure rises until around 50–60 years of age and then tends to decrease, while systolic pressure continues to rise. This pattern explains the high prevalence of isolated systolic hypertension in older adults, as evidenced by the results. This condition is related to the loss of arterial elasticity caused by structural and functional changes, such as calcium and collagen accumulation, rupture of elastin fibers, and endothelial dysfunction. These changes are exacerbated by conditions common in aging, such as diabetes, dyslipidemia, and atherosclerosis (28,29). From this perspective, measures for the prevention, control, and monitoring of systolic and diastolic blood pressure should be included at all stages of the life course of these vulnerable, marginalized, and often invisible individuals—especially from middle age to older adulthood—through clinical care practices, since hypertension is considered a cardiovascular disease for human health and can exacerbate clinical complications and morbidities and mortalities that require greater caution and priority in this population.

In a cross-sectional study conducted with 303 hypertensive patients in northeastern Brazil, it was highlighted that drug adherence is not a simple task, as it is influenced by a complex interaction of individual, cultural and socioeconomic factors, factors such as lack of regular access to health services, financial barriers and lack of information on the importance of lifestyle changes, which constitute significant obstacles to effective control of hypertension (30). 

Evidence indicates that lack of adherence to antihypertensive treatment can compromise blood pressure control, leading to increased levels (31,32). The SPRINT study revealed that patients with lower adherence to treatment had significantly higher systolic blood pressures compared to those who maintained high adherence (32). 

Longitudinal studies have shown that lower educational attainment is associated with higher systolic blood pressure levels, even after adjusting for other factors (33). This can be explained by less healthy habits more common in these groups, such as high sodium and alcohol consumption, low potassium intake, and higher body mass index (34). In the Quilombola population, this reality is aggravated by social, racial, and geographic inequalities, as well as deficiencies in the healthcare system, such as a lack of professional training and failures in medication distribution (35). Such data reinforce the need for specific policies and investments aimed at health equity (36). From this perspective, disparities in hypertension within Quilombola communities result from socioeconomic, behavioral, and environmental factors, requiring public health actions tailored to their specificities to ensure effective prevention and control.

It is acknowledged that this study has limitations, such as its cross-sectional design, self-reported data, and the absence of dietary information. Furthermore, its conclusions are limited to the Quilombola community studied. Nonetheless, its relevance prevails, as the results indicate low medication adherence, a high rate of inadequate blood pressure control, and elevated mean systolic and diastolic blood pressures. This underscores the need for culturally appropriate interventions to increase treatment adherence and improve cardiovascular health in this population.

The study reveals that more than half of the investigated Quilombola population has inadequate hypertension control, especially among individuals with type 2 diabetes, low medication adherence, lower educational attainment, and older age. These data highlight barriers to access to disease treatment and the absence of policies addressing the social determinants of health. Effective and culturally sensitive interventions are essential to improve blood pressure control and reduce disparities in this population.
