# Supplementary material for: Factors associated with inadequate blood pressure control among hypertensive individuals living in a Quilombola Community: a cross-setional study, Brazil, 2017-2018
Source: Epidemiol Serv Saude. 2025 Oct 3;34:e20240556. doi: 10.1590/S2237-96222025v34e20240556.en (PMC12494371; doi:10.1590/S2237-96222025v34e20240556.en)
Supplement: Translation

# Translation: Fatores associados ao controle pressórico inadequado em hipertensos residentes em comunidade quilombola: estudo transversal, Brasil, 2017-2018

**DOI:** 10.1590/S2237-96222025v34e20240556.pt

**Authors:** Randson Souza Rosa, Junior Santos Menezes, Edson Zangiacomi Martinez, Rita Narriman Silva de Oliveira Boery, Ícaro José Santos Ribeiro, Jean Carlos Zambrano Contreras, José de Bessa, Ricardo Franklin de Freitas Mussi, Sávio Luiz Ferreira Moreira, Gisele Silveira Lemos, Isleide Santana Cardoso Santos

**Affiliations:** 1 Universidade Estadual do Sudoeste da Bahia, Departamento de Saúde, Jequié, BA, Brasil; 2 Universidade Estadual de Feira de Santana, Departamento de Saúde, Feira de Santana, BA, Brasil; 3 Universidade de São Paulo, Faculdade de Medicina de Ribeirão Preto, Ribeirão Preto, SP, Brasil; 4 Universidade Estadual de Santa Cruz, Departamento de Saúde, Ilhéus, BA, Brasil; 5 Universidade do Estado da Bahia, Departamento de Educação, Caetité, BA, Brasil

**Keywords:** Pressão Arterial, Fatores de Risco, Quilombolas, População Negra, Estudos Transversais

## Abstract

**Objetivo:**

Identificar os fatores associados à prevalência do controle pressórico inadequado em hipertensos residentes em uma comunidade quilombola no Nordeste do Brasil.

**Métodos:**

Estudo epidemiológico de delineamento transversal com a participação de residentes do Quilombo do Barro Preto (Jequié, Bahia) com idade de 35 a 79 anos. O controle da pressão arterial foi classificado como inadequado para pressão arterial sistólica ≥140 mmHg e/ou pressão arterial diastólica ≥90 mmHg. A adesão medicamentosa foi avaliada pela Morisky Medication Adherence Scale. Utilizando-se regressão log-binomial, foram estimadas razões de prevalência (RP) ajustadas por sexo e por idade com intervalos de confiança de 95% (IC95%), para comparar diferenças entre as prevalências de controle pressórico inadequado entre grupos de interesse. Comparações entre médias basearam-se em testes t de Student e análise de variância.

**Resultados:**

Entre os 300 participantes, 71,7% eram mulheres, 49,3% se autodeclararam pretos, 41,0% pardos e 39,7% tinham nível fundamental incompleto. A pressão arterial sistólica associou-se com a idade, com média maior entre pessoas com mais de 65 anos, já a média da pressão arterial diastólica foi maior entre pessoas com até 55 anos. A prevalência de controle da pressão inadequada foi de 66,3% (IC95% 60,7; 71,7), sendo maior em pessoas com diabetes tipo 2 (RP 1,28; IC95% 1,09; 1,51) e baixa adesão a medicamentos (RP 1,27; IC95% 1,01; 1,59).

**Conclusões:**

A população quilombola apresenta dificuldades no controle da hipertensão arterial, com alta prevalência de descontrole pressórico, especialmente entre pessoas com diabetes tipo 2 e baixa adesão ao tratamento medicamentoso.

**Aspectos éticos**

A pesquisa respeitou os princípios éticos, obtendo os seguintes dados de aprovação:

Comitê de ética em pesquisa: Faculdade Independente do Nordeste

Número do parecer: 2.015.327

Data de aprovação: 13/4/2017

Certificado de apresentação de apreciação ética: 66705617.2.0000.5578

Registro do consentimento livre e esclarecido: Obtido de todos os participantes antes da coleta.

## Introdução

A hipertensão arterial é uma das doenças crônicas mais comuns no mundo e um grande desafio para a saúde pública. Em 2019, foi a principal causa de morte entre mulheres e a segunda entre homens, sendo responsável por cerca de 11 milhões de óbitos (1). Afrodescendentes são especialmente afetados, apresentando maiores taxas de prevalência e de morbimortalidade em relação a outros grupos étnico-raciais (2).

As comunidades afrodescendentes no Brasil vivenciam uma transição epidemiológica marcada pelo estilo de vida urbano, que eleva o risco de doenças cardiometabólicas, especialmente entre as mulheres, que apresentam uma combinação mais significativa de fatores de risco em comparação aos homens (3). Em Vitória da Conquista, Bahia, 45,4% dos moradores quilombolas apresentaram hipertensão, associada a fatores como idade, baixa escolaridade, classe econômica, insegurança no bairro, sedentarismo e obesidade (4). Pessoas negras, especialmente as de baixa renda, enfrentam maiores barreiras para o tratamento, como falta de suporte social, dificuldade de acesso a alimentos saudáveis e à prática de atividades físicas, além de limitações financeiras. A hipertensão não controlada eleva o risco de complicações graves e aumenta a sobrecarga nos sistemas de saúde dos países em desenvolvimento (5,6).

Evidências do estudo Reasons for Geographic and Racial Differences in Stroke (REGARDS) destacaram que determinantes sociais, como baixa renda, baixa escolaridade, áreas com escassez de profissionais de saúde e alta pobreza, aumentam o risco de pressão arterial descontrolada em adultos negros (7). O Estudo de coorte Bambuí-Epigen identificou que a posição socioeconômica - especialmente escolaridade e renda familiar - é o principal fator não biológico associado aos níveis de pressão arterial sistólica e ao controle da hipertensão, independentemente da idade, do sexo e de outros indicadores de saúde (8). Esse mesmo estudo evidenciou que o controle da pressão arterial em afrodescendentes é influenciado por fatores socioeconômicos, culturais, comportamentais e biológicos, sendo a posição socioeconômica - como baixa escolaridade e renda - mais determinante para o controle da hipertensão do que a própria ancestralidade genômica africana (8). Já o estudo de corte transversal de base populacional que utilizou dados do Projeto COMQUISTA (Comunidades Quilombolas de Vitória da Conquista) evidenciou que comunidades quilombolas apresentam alta prevalência de pré-hipertensão e hipertensão associadas ao sexo masculino, à baixa escolaridade, à diabetes e ao sobrepeso/à obesidade (9).

As comunidades quilombolas, geralmente localizadas em áreas rurais, são formadas por descendentes de africanos com forte herança cultural e histórica de resistência que compartilham saberes, práticas de saúde e modos de vida herdados dos antepassados (10). No entanto, enfrentam múltiplas vulnerabilidades, como baixa renda, falta de saneamento básico, moradias precárias, dificuldades de acesso a serviços de saúde e públicos, além de insegurança alimentar e nutricional (11).

O controle da hipertensão entre pessoas negras é desproporcionalmente afetado por fatores comportamentais, ambientais, sociais e estruturais, o que evidencia que o aumento da conscientização e das taxas de tratamento, embora importantes, não são suficientes. É essencial que as estratégias de saúde pública sejam adaptadas às necessidades específicas desses indivíduos e de suas comunidades (12). A baixa adesão ao tratamento é um dos principais desafios para o controle da pressão arterial entre pessoas negras, sendo a não adesão aos medicamentos anti-hipertensivos uma barreira significativa que contribui diretamente para as disparidades raciais na morbidade e na mortalidade cardiovasculares (13). Os estudos epidemiológicos são importantes para mapear as doenças que mais acometem as populações quilombolas, orientando de forma mais objetiva as políticas públicas para atender suas demandas específicas (14,15). 

A hipertensão arterial é uma das principais causas de morbimortalidade no Brasil, com impacto ainda mais acentuado em populações socialmente vulneráveis. No contexto das comunidades quilombolas - marcadas por desigualdades históricas, racismo estrutural e acesso limitado aos serviços de saúde - o controle pressórico inadequado representa um importante desafio para a saúde pública. Apesar da alta carga de doenças cardiovasculares entre afrodescendentes, há uma lacuna de estudos voltados especificamente para os fatores associados ao controle da hipertensão nessas comunidades, especialmente no Nordeste do Brasil.

O presente estudo se justifica pela necessidade de evidenciar resultados contextualmente relevantes sobre os determinantes do descontrole pressórico entre hipertensos quilombolas, considerando fatores individuais, socioeconômicos e fatores associados à saúde. A pesquisa é inovadora por integrar diferentes aspectos (faixa etária e adesão ao uso de medicamentos) em uma população historicamente invisibilizada nas políticas e nos estudos epidemiológicos tradicionais.

Ao identificar os principais fatores associados ao controle inadequado da pressão arterial nesse grupo, este estudo poderá subsidiar a formulação de estratégias mais equitativas e culturalmente sensíveis para o enfrentamento das doenças cardiovasculares em populações negras, contribuindo tanto para a redução das iniquidades em saúde quanto para a efetivação da Política Nacional de Saúde Integral da População Negra. Sendo assim, o objetivo deste artigo foi identificar os fatores associados à prevalência do controle pressórico inadequado em hipertensos residentes em uma comunidade quilombola no Nordeste do Brasil.

## Métodos

### Delineamento do estudo 

Trata-se de um estudo epidemiológico de delineamento transversal. 

### Contexto

O estudo foi realizado na Comunidade Quilombola do Barro Preto, situada no bairro Caixa D’Água, dentro da área de cobertura da Unidade Básica de Saúde Odorico Motta, localizada na zona urbana de Jequié, na região Sudoeste da Bahia.

A comunidade é reconhecida oficialmente como Comunidade Remanescente de Quilombola e foi certificada pela Fundação Cultural Palmares em 14 de fevereiro de 2007, sob o registro nº 01420.000313/2007-64. Esse reconhecimento foi reafirmado pela Portaria nº 104/2016, publicada no Diário Oficial da União em 20 de maio de 2016 (15).

A comunidade abrange 26 ruas e duas praças, possui aproximadamente 2.066 casas, onde residem cerca de 1.262 famílias, totalizando uma população de aproximadamente 7.130 pessoas. 

A escolha dessa comunidade justifica-se pelo fato de ela abrigar um contingente de pessoas quilombolas, que mantém saberes tradicionais, em uma região localizada em área urbana.

### Participantes

A população da pesquisa correspondeu ao número de registros de pessoas com diagnóstico de hipertensão arterial sistêmica cadastradas na Unidade de Saúde da Família da comunidade do Quilombo do Barro Preto, situada em Jequié, Bahia (400 pacientes hipertensos), em uso de anti-hipertensivos, de ambos os sexos, com idade entre 35 e 79 anos. Todos esses indivíduos são quilombolas autodeclarados, e as entrevistas foram feitas em seus domicílios por uma equipe previamente treinada. A pesquisa foi realizada no período entre novembro de 2017 e março de 2018.

### Instrumentos de pesquisa

O estudo utilizou questões do instrumento de “satisfação dos usuários hipertensos com os serviços da rede de atenção primária”, validado para uso em hipertensos adultos atendidos nos serviços do Brasil (16). Esse instrumento possui duas partes. A primeira, contém um espaço destinado para coleta de dados, como pressão arterial, dados antropométricos e sociodemográficos. A segunda parte aborda fatores de risco e comorbidades, cujas informações foram obtidas de modo autorreferido. Para avaliar a adesão medicamentosa, foi aplicada a versão em português da Morisky Medication Adherence Scale (MMAS-8, traduzida como Escala de Adesão Terapêutica de Morisky) adaptada e validada para a língua portuguesa (17). 

Do total de hipertensos quilombolas identificados, 25 foram excluídos inicialmente por ausência ou por mudança de residência. Após as entrevistas, mais 75 foram excluídos por não terem realizado a coleta sanguínea ou por terem preenchido os questionários de forma incompleta. Assim, a amostra final foi composta por 300 participantes, que atenderam aos critérios de elegibilidade e completaram todas as etapas da coleta de dados.

### Fonte de dados e mensuração

A primeira etapa da coleta de dados incluiu a aplicação dos questionários, seguida pela avaliação da composição corporal e pela aferição da pressão arterial para a avaliação clínica. A pressão arterial foi aferida duas vezes em ambiente tranquilo, sendo a primeira realizada após 10 minutos do início da avaliação clínica e a segunda cinco minutos depois, conforme as recomendações das Diretrizes Brasileiras de Hipertensão Arterial (18). Para a aferição, foi utilizado um esfigmomanômetro digital da marca Omron (modelo HEM-7113), com manguito ajustado de acordo com o tamanho e o formato do braço do paciente, validado e recomendado pela Sociedade Brasileira de Hipertensão (19). Nessas aferições, os participantes esvaziaram a bexiga e posicionaram o braço esquerdo na altura do coração. Todas as medições foram realizadas por profissionais previamente treinados para atuar na coleta domiciliar. Para a análise dos dados, foi considerada a média entre a primeira e a segunda aferição da pressão arterial.

Quanto à mensuração da composição corporal, incluindo peso (em kg), estatura (em cm) e circunferência da cintura (em cm), foram utilizadas técnicas padronizadas de avaliação antropométrica. Foi usada uma balança de bioimpedância digital da marca Omron (modelo HBF-514C) para avaliação da massa corporal, com os participantes trajando roupas leves e posicionados no centro do aparelho. Para medir a estatura, foi utilizado o estadiômetro portátil personal Capriche da marca Sanny (modelo ES2060), com um campo de medição de 210 cm. Os participantes estavam descalços e com os pés justapostos. A avaliação da circunferência da cintura foi realizada com uma fita métrica inelástica e extensível da marca Sanny (modelo SN-4010), sem trava, com os participantes em posição supina.

Foram adotados os pontos de corte para predição de obesidade de circunferência da cintura igual ou superior a 102 cm para homens, e igual ou superior a 88 cm para mulheres, conforme estabelecido pelos critérios do National Cholesterol Education Program III (20). A partir das medidas de peso e de estatura, a classificação de sobrepeso e de obesidade foi determinada pelo índice de massa corporal, calculado a partir da divisão do peso pela estatura ao quadrado. De acordo com as diretrizes da Organização Mundial da Saúde de 1995, os participantes foram classificados como apresentando excesso de peso (sobrepeso e obesidade) quando o índice de massa corporal foi igual ou superior a 25 kg/m^2^, e como eutróficos ou normais quando inferior a 25 kg/m^2^ (21).

A segunda etapa foi conduzida na Unidade de Saúde da Família local, onde técnicos treinados cedidos pelo laboratório parceiro da Secretaria Municipal de Saúde coletaram sangue para análise do perfil lipídico dos participantes do estudo.

### Variáveis 

Para a análise dos dados, a variável dependente foi o controle da pressão arterial, classificada em pressão arterial não controlada (inadequada) quando a pressão arterial sistólica foi maior ou igual a 140 mmHg e/ou a pressão arterial diastólica foi maior ou igual a 90 mmHg, e o controle da pressão arterial adequado, indicado por leituras de pressão arterial inferiores a 140/90 mmHg (19). As variáveis independentes foram: sexo (masculino ou feminino), faixa etária (até 55 anos, 56-65 anos ou mais de 65 anos), raça/cor da pele (preta, parda, branca, amarela ou indígena), escolaridade (analfabeto, alfabetizado, fundamental incompleto, fundamental completo, médio incompleto, médio completo ou superior incompleto), situação familiar/conjugal (mora com companheiro(a), mora com parentes ou vive só), diabetes melitos tipo 1 (sim, não ou não sabe), diabetes tipo 2 (sim, não ou não sabe), tabagismo (sim ou não), etilismo (sim, não ou não respondeu), inatividade física (sim ou não), excesso de peso (não; sim; não respondeu), infarto agudo do miocárdico (sim ou não), outras coronopatias (sim, não ou não sabe), acidente vascular cerebral (sim ou não), e doença renal crônica (sim, não ou não sabe). 

A MMAS-8 é composta por oito itens e a cada um são oferecidas as opções de resposta: “nunca”, “às vezes”, “frequentemente” e “sempre”. A adesão terapêutica é, então, avaliada pela soma das respostas “não” (1 ponto cada). As classificações de adesão são: alta (oito pontos), média (6 a <8 pontos) e baixa (<6 pontos).

### Controle de viés

As variáveis independentes foram coletadas de forma autorreferida. Portanto, pode-se ter o viés de esquecimento e o da falta de compreensão ou de entendimento sobre o questionamento realizado ou sobre o diagnóstico de alguma comorbidade.

### Métodos estatísticos

Para a descrição dos dados, foram obtidas as médias e os desvios-padrão para as variáveis quantitativas, e as frequências absolutas (n) e relativas (%) para as variáveis categóricas. O percentual de pessoas com controle de pressão inadequado foi estimado com intervalos de confiança 95% (IC95%), segundo o método de Clopper-Pearson (22), implementado no pacote DescTools do programa R, versão 4.3.2. Razões de prevalência (RP) ajustadas por sexo e por idade foram usadas para medir a associação entre o controle de pressão arterial inadequado e as variáveis independentes. Essas medidas e os seus correspondentes IC95% foram estimados por modelos de regressão log-binomial (23), considerando sexo e idade como variáveis de confusão. O pacote logbin do programa R foi usado para o ajuste desses modelos (23).

O teste t de Student para amostras independentes foi usado para comparações entre as médias das idades das pessoas classificadas em pressão arterial não controlada e controlada. A análise de variância (com testes baseados em somas de quadrados tipo III) foi usada para comparar as médias populacionais de pressão arterial sistólica e de pressão arterial diastólica entre diferentes subgrupos, tendo a idade e o sexo como covariáveis. 

Esses procedimentos consideraram um nível de significância de 0,05 e foram adequados à presença ou à ausência de homoscedasticidade (verificada pelo teste de Bartlett), já pressupostos de normalidade de resíduos, simetria e efeitos de valores atípicos foram verificados por gráficos apropriados (boxplots e gráficos de probabilidade normal). O banco de dados foi armazenado no Mendeley Data (24).

## Resultados

O controle de pressão inadequado foi prevalente em (n=199; 66,3%) (IC95% 60,7; 71,7) dos participantes. A distribuição das idades dos participantes com controle da pressão inadequado e adequado foi semelhante, com médias de 60,3 anos e 59,5 anos, respectivamente (desvios-padrão (DP) iguais a 11,2 anos e 10,7 anos, respectivamente, sem evidências de diferenças entre as médias populacionais, com p-valor =0,566, segundo teste t de Student). (Tabela 1).

**Tabela 1:** Razões de prevalências e intervalos de confiança de 95% (IC95%) ajustados por sexo e por idade do controle da pressão inadequado pelas variáveis do estudo. Brasil, 2018 (n=300)

		Controle da pressão inadequado		Razão de prevalência ^a^
Variáveis	n (%)	n	% (IC95%)	% (IC95%)
Total	300 (100)	199	66,3 (60,7; 71,7)	-
Sexo				
Feminino	215 (71,7)	140	65,1 (58,3; 71,5)	Referência
Masculino	85 (28,3)	59	69,4 (58,5; 79,0)	1,03 (0,86; 1,22)
**Faixa etária** (ano)				
Até 55	106 (35,4)	73	68,9 (59,1; 77,5)	Referência
56-65	94 (31,2)	54	57,4 (46,8; 67,6)	0,84 (0,67; 1,04)
Mais de 65	100 (33,4)	72	72,0 (62,1; 80,5)	1,04 (0,87; 1,24)
**Raça/cor da pele**				
Preta	148 (49,4)	101	68,2 (60,1; 75,6)	Referência.
Parda	123 (41,0)	80	65,0 (55,9; 73,4)	0,95 (0,81; 1,13)
Branca	24 (8,0)	15	62,5 (40,6; 81,2)	0,87 (0,63; 1,21)
Amarela	4 (1,3)	2	50,0 (6,8; 93,2)	^b^
Indígena	1 (0,3)	1	-	^b^
Escolaridade				
Analfabeto	88 (29,3)	65	73,9 (63,4; 82,7)	Referência
Alfabetizado	38 (12,7)	27	71,1 (54,1; 84,6)	0,96 (0,76; 1,22)
Fundamental incompleto	119 (39,8)	75	63,0 (53,7; 71,7)	0,84 (0,69; 1,02)
Fundamental completo	25 (8,3)	15	60,0 (38,7; 78,9)	0,74 (0,51; 1,06)
Médio incompleto	12 (4,0)	8	66,7 (34,9; 90,1)	0,83 (0,54; 1,28)
Médio completo	16 (5,3)	9	56,2 (29,9; 80,2)	0,71 (0,45; 1,28)
Superior completo	2 (0,6)	0	-	^b^
**Situação familiar**/conjugal				
Mora com companheiro(a)	176 (58,7)	117	66,5 (59,0; 73,4)	Referência
Mora com parentes	96 (32,0)	59	61,5 (51,0; 71,2)	0,93 (0,77; 1,13)
Vive só	28 (9,3)	23	82,1 (63,1; 93,9)	1,16 (0,93; 1,45)
Tabagismo				
Não	272 (90,7)	179	65,8 (59,8; 71,4)	Referência
Sim	28 (9,3)	20	71,4 (51,3; 86,8)	1,11 (0,87; 1,41)
Etilismo				
Não	248 (82,7)	165	66,5 (60,3; 72,4)	Referência
Sim	48 (16,0)	31	64,6 (49,5; 77,8)	0,93 (0,73; 1,18)
Não responderam	4 (1,3)	3	-	-
**Inatividade física**				
Não	150 (50,0)	101	67,3 (59,2; 74,8)	Referência
Sim	150 (50,0)	98	65,3 (57,1; 72,9)	0,96 (0,82; 1,13)
**Adesão medicamentosa**				
Alta	73 (24,3)	42	57,5 (45,4; 69,0)	Referência;
Média	117 (39,0)	76	65,0 (55,6; 73,5)	1,12 (0,89; 1,41)
Baixa	110 (36,7)	81	73,6 (64,4; 81,6)	1,27 (1,01; 1,59)
**Diabetes tipo 1**				
Não	211 (70,3)	145	68,7 (62,0; 74,9)	Referência
Sim	6 (2,0)	4	66,7 (22,3; 95,7)	0,91 (0,51; 1,61)
Não sabem	83 (27,7)	50	60,2 (48,9; 70,8)	0,88 (0,73; 1,07)
**Diabetes tipo 2**				
Não	147 (49,0)	92	62,6 (54,2; 70,4)	Referência
Sim	71 (23,7)	58	81,7 (70,7; 89,9)	1,28 (1,09; 1,51)^d^
Não sabem	82 (27,3)	49	59,8 (48,3; 70,4)	0,96 (0,78; 1,19)
**Excesso de peso**				
Não	101 (33,7)	69	68,3 (58,3; 77,2)	Referência
Sim	193 (64,3)	127	65,8 (58,6; 72,5)	0,99 (0,84; 1,78)
Não responderam	6 (2,0)	3	-	
**Infarto agudo do miocárdio**				
Não	293 (97,7)	194	66,2 (60,5; 71,6)	Referência
Sim	7 (2,3)	5	71,4 (29,0; 96,3)	1,09 (0,66; 1,79)
**Outra coronariopatia**				
Não	272 (90,7)	185	68,0 (62,1; 73,5)	Referência
Sim	26 (8,7)	13	50,0 (29,9; 70,1)	0,74 (0,50; 1,09)
Não sabem	2 (0,6)	1	-	
**Acidente vascular cerebral**				
Não	284 (94,7)	189	66,5 (60,7; 72,0)	Referência
Sim	16 (5,3)	10	62,5 (35,4; 84,8)	0,90 (0,59; 1,36)
**Doença renal crônica**				
Não	204 (68,0)	133	65,2 (58,2; 71,7)	Referência
Sim	20 (6,7)	14	70,0 (45,7; 88,1)	1,07 (0,79; 1,44)
Não sabem	76 (25,3)	2	-	-

^a^Razões de prevalências ajustada por sexo e por idade; ^b^Não estimado devido ao tamanho amostral reduzido na classe correspondente.

Como pode ser verificado na Tabela 1, o perfil sociodemográfico da amostra foi composto por maior proporção de pessoas: do sexo feminino (n=215; 71,7%), que se autodeclararam pretas (n=148; 49,3%) e pardas (n=123; 41,0%), com faixa etária até os 55 anos (n=106; 35,4%), com escolaridade de nível fundamental incompleto (n=119; 39,7%) e que moram com o(a) companheiro(a) (n=176; 58,7%). 

Quanto ao estilo de vida (Tabela 1), o tabagismo foi relatado por 28 participantes (9,2%), 48 participantes (16%) informaram consumir bebidas alcoólicas, 150 participantes (50,0%) declararam ser inativos fisicamente, enquanto 110 participantes (36,7%) e 117 participantes (39%) apresentaram, respectivamente, baixa e média adesão medicamentosa.

No que concerne às comorbidades (Tabela 1) 6 participantes (2,0%) reportaram diabetes tipo 1,71 participantes (23,7%) reportaram diabetes tipo 2, o excesso de peso foi prevalente em 193 participantes (64,3%), o infarto em 7 participantes (2,3%), outra coronariopata em 26 participantes (8,7%), acidente vascular cerebral em 16 participantes (5,3%) e doença renal crônica em 20 participantes (6,7%).

Considerando toda a amostra, as médias da pressão arterial sistólica e as da pressão arterial diastólica foram 150,3 mmHg, com desvio-padrão (DP) de 24,8 mmHg e de 86,4 mmHg (DP de 13,3 mmHg), respectivamente (Tabela 2).

**Tabela 2:** Valores médios e desvios-padrão (DP) da pressão arterial sistólica e da diastólica de acordo com variáveis do estudo. Brasil, 2018 (n=300)

		Pressão arterial sistólica (mmHg)		Pressão arterial diastólica (mmHg)	
Variáveis	n	Média (DP)	p-valor^a^	Média (DP)	p-valor^a^
Total	300	150,3 (24,8)	-	86,4 (13,3)	-
Sexo			0,920		0,623
Feminino	215	150,2 (25,3)		86,4 (13,2)	
Masculino	85	150,3 (23,4)		86,6 (13,7)	
**Faixa etária** (ano)			0,019		<0,001
Até 55	106	150,6 (26,1)		92,3 (14,3)	
56-65	94	145,0 (23,8)		83,1 (12,1)	
Mais de 65	100	154,9 (23,4)		83,3 (11,2)	
**Raça/cor da pele**			0,886		0,59
Preta	148	150,7 (26,1)		87,3 (14,8)	
Parda	123	149,5 (23,4)		86,1 (11,8)	
Branca	24	150,4 (25,1)		84,4 (12,3)	
Amarela	4	153,3 (19,7)		82,0 (6,0)	
Escolaridade			0,020		0,453
Analfabeto	88	158,2 (25,8)		85,3 (13,4)	
Alfabetizado	38	152,6 (28,1)		84,4 (12,1)	
Fundamental incompleto	119	146,7 (22,8)		87,5 (13,2)	
Fundamental completo	25	146,8 (18,1)		87,2 (11,3)	
Médio incompleto	12	140,7 (23,9)		90,7 (16,2)	
Médio completo	16	143,6 (26,0)		87,2 (17,1)	
Superior completo	2	128,3 (8,8)		70,8 (13,8)	
**Situação familiar**/conjugal			0,529		0,698
Mora com companheiro(a)	176	149,0 (24,0)		87,0 (12,6)	
Mora com parentes	96	151,0 (24,3)		85,5 (14,6)	
Vive só	28	156,4 (26,0)		86,3 (13,3)	
Tabagismo			0,864		0,794
Não	272	150,2 (25,1)		86,5 (13,3)	
Sim	28	151,2 (21,8)		85,6 (13,8)	
Etilismo			0,188		0,382
Não	248	151,1 (24,7)		85,8 (13,2)	
Sim	48	145,2 (23,7)		89,4 (14,0)	
**Inativo fisicamente**			0,646		0,442
Não	150	150,7 (24,2)		87,4 (13,4)	
Sim	150	149,9 (25,4)		85,5 (13,3)	
**Adesão medicamentosa**			0,015		0,032
Baixa	110	154,3 (26,5)		89,8 (14,4)	
Média	117	149,8 (24,5)		85,2 (12,4)	
Alta	73	145,1 (21,5)		83,4 (12,1)	
**Diabetes tipo 1**			0,451		0,789
Não	211	151,2 (24,8)		86,7 (12,8)	
Sim	6	158,1 (24,7)		84,2 (9,4)	
Não sabem	83	147,5 (24,6)		85,8 (14,8)	
**Diabetes tipo 2**			0,081		0,738
Não	147	149,1 (24,6)		86,6 (13,4)	
Sim	71	156,0 (24,5)		86,9 (11,2)	
Não sabem	82	147,3 (24,7)		85,8 (14,9)	
**Excesso de peso**			0,162		0,267
Não	101	153,3 (24,7)		87,1 (13,2)	
Sim	193	148,7 (24,4)		86,2 (13,5)	
**Infarto agudo do miocárdio**			0,998		0,441
Não	293	150,3 (24,7)		86,6 (13,3)	
Sim	7	151,1 (30,1)		81,4 (15,4)	
**Outra coronariopatia**			0,064		0,074
Não	272	151,2 (24,9)		86,8 (13,5)	
Sim	26	141,4 (22,2)		82,7 (10,6)	
**Acidente vascular cerebral**			0,745		0,287
Não	284	150,1 (24,3)		86,3 (13,1)	
Sim	16	152,8 (31,3)		88,9 (16,9)	
**Doença renal crônica**			0,982		0,803
Não	204	149,3 (24,7)		85,7 (13,6)	
Sim	20	149,3 (22,6)		86,5 (10,5)	

^a^p-valor obtido da análise de variância (testes tipo III), com ajuste por sexo e por idade.

Foi identificada diferença significativa na média da pressão arterial sistólica e da diastólica conforme a faixa etária (Tabela 2). Nesse sentido, a média da pressão arterial sistólica foi maior no grupo das pessoas com mais de 65 anos, enquanto a média da pressão arterial diastólica foi maior entre aquelas com menos de 55 anos. 

## Discussão

Este estudo evidencia a vulnerabilidade de uma comunidade quilombola ao identificar que dois terços das pessoas com hipertensão apresentam controle da pressão inadequado. A frequência de pessoas nessa condição é semelhante entre os sexos, não mostra associação com a idade e se mostra elevada mesmo entre pessoas com maior escolaridade. Isso reforça a importância do Sistema Único de Saúde e de políticas públicas de assistência farmacêutica, dado que um quantitativo significativo de pessoas nas comunidades quilombolas vive em condições economicamente desfavoráveis e, em diversas ocasiões, não possui rendimento suficiente para manutenção do uso das medicações sem que sejam fornecidas pelo sistema público (12). 

Entretanto, mesmo entre as pessoas com alta adesão ao uso de medicamentos, é observada uma frequência relativamente elevada de controle inadequado da pressão (57,5%). As intervenções não farmacológicas no estilo de vida devem ser encorajadas entre os não-hipertensos como cuidados preventivos para o desenvolvimento da doença e, para a hipertensão estabelecida, como terapia adjuvante, a fim de atenuar o risco de desenvolver outras morbidades e reduzir a necessidade de medicamentos (25). Essas intervenções incluem a prática regular de atividade física, o controle do peso, a cessação do tabagismo, a redução do estresse e a prevenção do consumo excessivo de álcool (26). 

Embora fatores como tabagismo, etilismo, excesso de peso e inatividade física, não tenham mostrado associação direta com o controle da pressão arterial, a alta prevalência de excesso de peso e de inatividade física sugerem a necessidade de monitoramento e de intervenções de saúde. Além disso, boa parte dos participantes relataram diabetes tipo 2 com maior frequência de controle inadequado da pressão. A gestão de múltiplos aspectos da saúde em pessoas portadoras de diabetes pode dificultar a adesão aos tratamentos, destacando a importância de boas práticas clínicas para reduzir complicações e melhorar a qualidade de vida. A hipertensão arterial e o diabetes são consideradas corresponsáveis pelas principais causas de mortalidade e de hospitalizações no Brasil (27), e a coexistência do diabetes tipo 2 com a hipertensão pode complicar ainda mais o manejo da pressão arterial, exigindo um regime terapêutico mais rigoroso e complexo. 

A pressão arterial se eleva com a idade de forma não linear: a diastólica aumenta até os 50-60 anos e depois tende a diminuir, enquanto a sistólica continua subindo. Esse padrão explica a alta prevalência de hipertensão sistólica isolada em idosos, como evidenciada nos resultados. Tal condição está relacionada à perda da elasticidade das artérias, causada por alterações estruturais e funcionais, como acúmulo de cálcio e de colágeno, ruptura de fibras de elastina e disfunção endotelial. Essas mudanças são agravadas por condições comuns no envelhecimento, como diabetes, dislipidemia e aterosclerose (28,29). Nessa perspectiva, medidas de prevenção, de controle e de monitoramento da pressão arterial sistólica e da diastólica devem ser incluídas em todas as fases do curso de vida dessas pessoas consideradas vulnerabilizadas, marginalizadas e invisíveis, especialmente no curso de vida da meia idade para idosos, através de práticas de cuidados clínicos, uma vez que a hipertensão arterial é considerada uma doença cardiodeletéria para a saúde humana e pode exacerbar complicações clínicas e morbimortalidades que devem ser consideradas com mais cautela e prioridade nessa população.

Em um estudo transversal, realizado com 303 hipertensos no Nordeste do Brasil, destacou-se que a adesão medicamentosa não é uma tarefa simples, pois é influenciada por uma complexa interação de fatores individuais, culturais e socioeconômicos, fatores como a falta de acesso regular a serviços de saúde, as barreiras financeiras e a falta de informações sobre a importância de mudanças no estilo de vida, os quais constituem obstáculos significativos para o controle eficaz da hipertensão (30). 

Evidências indicam que a falta de adesão ao tratamento anti-hipertensivo pode comprometer o controle da pressão arterial, levando ao aumento de seus níveis (31,32). O estudo SPRINT revelou que pacientes com menor adesão ao tratamento apresentaram pressões arteriais sistólicas significativamente mais elevadas em comparação com aqueles que mantiveram alta adesão (32). 

Estudos longitudinais mostraram que menor escolaridade está associada a maiores níveis de pressão arterial sistólica, mesmo após ajustes para outros fatores (33). Isso pode ser explicado por hábitos menos saudáveis mais comuns nesses grupos, como alto consumo de sódio e de álcool, baixa ingestão de potássio e maior índice de massa corporal (34). Na população quilombola, essa realidade é agravada por desigualdades sociais, raciais e geográficas, além de deficiências no sistema de saúde, como falta de capacitação profissional e falhas na distribuição de medicamentos (35). Tais dados reforçam a necessidade de políticas específicas e de investimentos voltados à equidade em saúde (36). Nessa perspectiva, as disparidades na hipertensão em comunidades quilombolas resultam de fatores socioeconômicos, comportamentais e ambientais, demandando ações de saúde pública adaptadas às suas especificidades para que a prevenção e o controle sejam eficazes.

Reconhece-se que este estudo tem limitações, como o desenho transversal, dados autorrelatados e a ausência de informações sobre dieta. Além disso, suas conclusões são restritas à comunidade quilombola analisada. Mesmo assim, a sua relevância se sobrepõe, já que os resultados indicam baixa adesão a medicamentos, alto índice de controle inadequado da pressão arterial e médias elevadas de pressão sistólica e de diastólica. Isso reforça a necessidade de intervenções culturalmente adequadas para aumentar a adesão ao tratamento e melhorar a saúde cardiovascular dessa população.

O estudo revela que mais da metade da população quilombola investigada apresenta controle inadequado da hipertensão, especialmente entre pessoas com diabetes tipo 2, com baixa adesão a medicamentos, com menor escolaridade e idosas. Esses dados evidenciam barreiras no acesso ao tratamento de doenças e ausência de políticas que enfrentem os determinantes sociais da saúde. Intervenções eficazes e culturalmente sensíveis são essenciais para melhorar o controle da pressão arterial e reduzir as desigualdades nessa população.
